# New Insights Into the Virus-to-Prokaryote Ratio (VPR) in Marine Sediments

**DOI:** 10.3389/fmicb.2020.01102

**Published:** 2020-05-29

**Authors:** Miao Wei, Kuidong Xu

**Affiliations:** ^1^Laboratory of Marine Organism Taxonomy and Phylogeny, Institute of Oceanology, Chinese Academy of Sciences, Qingdao, China; ^2^University of Chinese Academy of Sciences, Beijing, China; ^3^Laboratory for Marine Biology and Biotechnology, Pilot National Laboratory for Marine Science and Technology, Qingdao, China; ^4^Center for Ocean Mega-Science, Chinese Academy of Sciences, Qingdao, China

**Keywords:** viral abundance, prokaryotic abundance, virus-to-prokaryote ratio, virus–host relationship, marine sediment

## Abstract

The virus-to-prokaryote ratio (VPR), which reflects the numerical dominance of viruses over their hosts, has been proposed as a proxy for assessing the relationship between viruses and prokaryotes. Previous studies showed that VPR values fluctuate over six orders of magnitude within and across various benthic ecosystems, with an average value of approximately 10. We hypothesize that this high VPR value is largely due to the inaccurate enumeration of viruses and prokaryotes (e.g., centrifugation treatments may lead to a three–fourfold overestimation of VPR). In this study, we evaluated the impact of processing methods on the determination of VPR values. Using an optimized procedure, we investigated the marine benthic VPR at 31 sites, from intertidal zones through continental shelves to abyssal plains, and assessed its monthly variation in two contrasting intertidal habitats (muddy-sand and sandy). By compiling 135 VPR data points of surface sediments from 37 publications, we reveal the effect of centrifugation on published VPR values and describe the spatial distribution of VPR values on a larger scale based on reliable data. The results showed that the commonly used centrifugation method may result in an overestimation of VPR values that are approximately one order of magnitude higher than those obtained using the dilution method, while other processing steps had a limited impact on the VPR. Our analysis indicates that the benthic VPR value is low and less varied across temporal and spatial scales, fluctuating mostly within 10, and the average VPR is approximately 2 in both marine and freshwater habitats. An insignificant seasonal pattern in the VPR was observed in the intertidal zone, with lower VPR values occurring at high temperatures. The VPR spatial distribution was primarily associated with sediment phaeophytin a, suggesting that the trophic conditions of the upper water column and the sedimentation of organic matter to the bottom are the key factors affecting VPR values. The mean VPR in benthic habitats is approximately one order of magnitude lower and much less varied than that observed in pelagic habitats, indicating that the virus–host relationship and the ecological function of viruses in the two ecosystems may be very different.

## Introduction

Viruses are recognized as being ubiquitous components in all aquatic environments, most of which are prokaryote-infecting viruses known as phages or cyanophages (Danovaro et al., 2008b). By lysing their hosts, viruses are capable of converting microbial biomass into dissolved organic matter, thus diverting it away from higher trophic levels (Corinaldesi et al., 2012). The viral-induced alterations in organic matter flows have been termed the ‘viral shunt’ (Wilhelm and Suttle, 1999; Danovaro et al., 2008b). Viruses have been suggested to have profound effects on biogeochemical cycling, microbial loop dynamics, and host diversity through the interactions with their hosts (Fuhrman, 1999; Wilhelm and Suttle, 1999; Wommack and Colwell, 2000; Weinbauer and Rassoulzadegan, 2004; Hambly and Suttle, 2005).

The virus-to-prokaryote ratio (VPR) is the proportion between the viral abundance (VA) and prokaryotic abundance (PA), which is thought to represent a balance between viral production and viral decay in a presumed steady-state (Maranger and Bird, 1995). As viral production is regulated by the metabolic activity of the prokaryotes (Glud and Middelboe, 2004), the VPR has been considered to be important information that reflects the virus–host relationship (Wommack and Colwell, 2000; Parikka et al., 2016). The VPR has also been used to compare the relative viral activity in different samples (Ogunseitan et al., 1990; Wommack et al., 1992). High VPR values are typically interpreted as high viral activity, while low values are attributed to low viral production or a high viral decay rate (Middelboe and Glud, 2006; Parikka et al., 2016). Therefore, the VPR is considered to be the basic index used to describe the relationship between viruses and prokaryotes and is important in understanding the role of viruses in the environment.

The accurate determination of VA and PA is a prerequisite for obtaining correct VPR values. Fluorescence microscopy (EFM) and flow cytometry (FCM) are both common methods used to enumerate viruses and prokaryotes in marine samples. Compared with EFM, FCM is more sensitive to fluorescent stain and less influenced by the skills of the operators (Brussaard, 2004) and thus has been commonly used for counting viruses and prokaryotes in water samples (e.g., Marie et al., 1999; Chen et al., 2001; Zhao et al., 2020). However, FCM is not widely applied for the determination of sediment VA and PA. To avoid instrument clogging, sediment samples must be centrifuged before determined by FCM, such a process could lead to underestimation and higher coefficient of variation of the enumeration (Glud and Middelboe, 2004; Siem-Jørgensen et al., 2008; Dai, 2012; Frossard et al., 2016). Currently, EFM is still the most widely used procedure for counting sediment viruses and prokaryotes, and it has been continuously optimized in the past decades of this century (Danovaro et al., 2001; Fischer et al., 2005; Helton et al., 2006; Dell’Anno et al., 2009; Danovaro and Middelboe, 2010; Suttle and Fuhrman, 2010).

Despite the efforts to improve the use of EFM to count sediment VA and PA, inconsistencies in the processing of samples among different researchers remained. For example, the concentrations of sodium pyrophosphate used in the treatment of samples were not uniform, and there is a controversy as to whether an ice bath should be performed when the sample is sonicated (Duhamel and Jacquet, 2006; Danovaro and Middelboe, 2010). The impacts of these different treatments on VPR values are still not known. The preservation of sediment samples has also a crucial impact on VA and PA determinations. The currently accepted effective preservation methods for sediment samples are to snap freeze and store the untreated/aldehyde-fixed sediments at −80°C/liquid nitrogen (hereafter referred to as ‘−80°C preservation’), or to process them immediately after sampling and store the slides at −20°C for a period of time (hereafter referred to as ‘slide preservation’) (Dell’Anno et al., 2009; Danovaro and Middelboe, 2010; Suttle and Fuhrman, 2010). However, the effect of −80°C preservation has never been assessed for benthic viruses, nor have the impact of −80°C or slide preservation on VPR values been evaluated.

The prevailing view is that viruses outnumber their prokaryotic hosts by an order of magnitude (Suttle, 2005; Danovaro et al., 2008b). By collecting and analyzing the data from 210 publications, Parikka et al. (2016) showed that the VPR value in pelagic habitats was on average 21.9 and approximately 10 in benthic habitats (12.1 in marine sediments and 9.2 in freshwater sediments), fluctuating over 6 orders of magnitude in both habitats. However, there are potential errors in these analyses, because some reported VPR values were biased due to the sample processing method. Glud and Middelboe (2004) noted that VPR values may be overestimated when the samples are centrifuged due to the lower extraction efficiency of prokaryotes than that of viruses. Siem-Jørgensen et al. (2008) compared the VPR values obtained by centrifugation and dilution and proposed that the VA and PA obtained by centrifugation should be corrected by a factor of 2.2 and 7.7, respectively. This result would indicate that the VPR would be overestimated by approximately 3.5-fold when using the centrifugation method. However, the deviation caused by centrifugation has not received enough attention, as centrifugation was still used in a number of subsequent studies. These problematic VPR values, which were used in the overall statistical analysis without correction, would greatly affect the overall assessment and correlation analyses of VPR values.

We hypothesize that the high value for the VPR is largely due to the inaccurate enumeration of viruses and prokaryotes caused by sample processing methods. In this study, we evaluated the impact of the primary steps of the sediment sample xtraction procedure and two preservation methods on VPR values. By using the optimized protocol, we analyzed the sediment samples collected from 31 sites from the intertidal zone through the continental shelf and to the abyssal plain, including monthly samplings throughout 1 year at sandy and muddy-sand sites, to explore the temporal and spatial distribution patterns of VA, PA, and VPR and to analyze their relationships with environmental factors. By collecting 135 reported VPR data points from 37 publications covering surface sediments of marine, freshwater and extreme environments, we further reveal the impact of sample processing methods on benthic VPR values and uncovered the values and fluctuations of benthic viruses in different habitats.

## Materials and Methods

### Study Sites and Sample Collection

**FIGURE 1:**
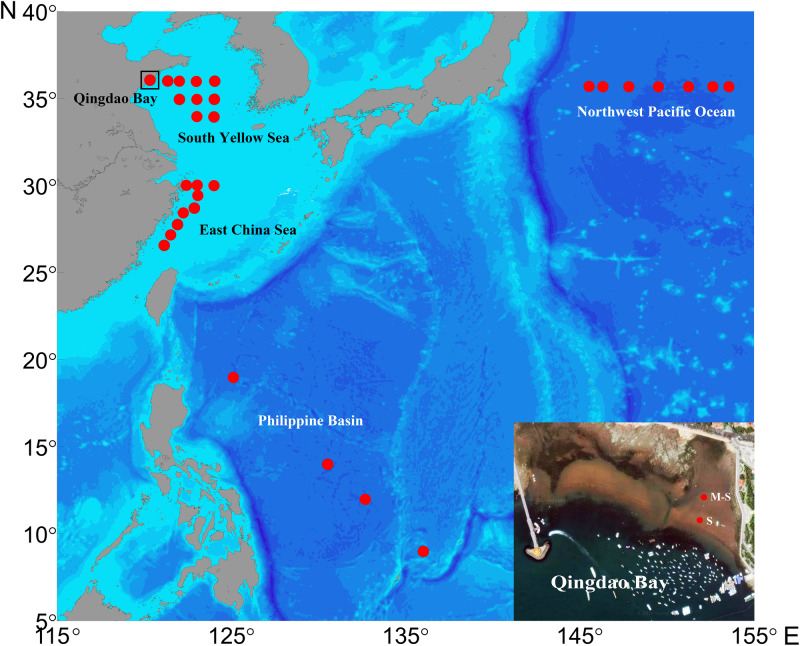
The location of the study sites. The inset shows the sandy (S) and muddy-sand (M-S) sites in the intertidal zone of the Qingdao Bay in the Yellow Sea.

Sediment samples were collected from five sea areas (Figure 1): (1) sandy and muddy-sand sites located approximately 100 m apart in the intertidal zone of Qingdao Bay on the China coast of the Yellow Sea (36°03′N, 120°19′E), where monthly samplings were conducted from January to December in 2016; (2) nine sites on the continental shelf of the south Yellow Sea (34–36°N, 121–124°E; mean depth of 64 m) from January 14 to 20, 2016; (3) nine sites on the East China Sea continental shelf (26–30°N, 121–123°E; mean depth of 66 m) from September 23 to October 01, 2016; (4) four sites on the deep-sea plain of the Philippine Basin in the tropical Western Pacific Ocean (9–19°N, 125–135°′E; mean depth of 5,141 m) from November 25 to December 29, 2015; and (5) seven sites on the deep-sea plain of the Northwest Pacific Ocean (35.5°N, 145–154°′E; mean depth of 5,811 m) during March 2017.

The intertidal sediments were collected during ebb tide. The shelf sea sediments were collected using a 0.1-m^2^ Gray-O′Hara box corer, and the deep-sea sediments were collected using a 0.25-m^2^ box corer. Samples of approximately 50 mL were scraped from the top 1 cm of undisturbed sediment with a sterile spoon and adequately homogenized. Subsamples (0.5 mL) were collected from the homogenized sediment and immediately fixed with 4.2 mL of 0.02-μm filtered seawater containing 2% formalin. The intertidal zone and Philippine Basin samples were processed immediately after collection, while those from the south Yellow Sea, East China Sea and Northwest Pacific Ocean were snap-frozen and stored in liquid nitrogen for 2 weeks, 2 months, and 3 months, respectively, until further processing. The rest of the homogenized sediments were placed in sterile sealed bags and stored at −20°C for subsequent measurements of environmental factors.

### Measurements of Environmental Factors

At each sampling site, the water content of the sediment was determined as the percentage of weight loss after drying the sediment at 60°C for 48 h. The concentrations of the sediment chlorophyll *a* (Chla) and phaeophytin *a* (Pha) were determined using a fluorescence spectrophotometer (Turner Designs Trilogy, United States) (Mantoura and Llewellyn, 1983). The total organic carbon (TOC) content of the sediment was measured using a Vario TOC Cube (Elementar, Germany) (Gaudette et al., 1974). The median grain size (MGS) of the sediment was measured using a particle-size analyzer (Cilas 1190). The temperature and salinity of the surface sediment in the intertidal zone was measured *in situ* with a thermometer and a hand-held refractometer, respectively, while the water depth, bottom water temperature and salinity in the continental shelf and deep seas were measured using a SeaBird CTD system. The bottom water temperature and salinity of the North Pacific sites were represented by one site in the North Pacific Ocean (160°E, 35°N; water depth of 4,330 m).

### Evaluation of the Extraction and Storage Procedures

The extraction and storage procedures were evaluated for the following conditions: (i) the final concentration of sodium pyrophosphate, where the sediments were incubated in the dark with 0.5, 2, 3, 5, and 7 mmol/L sodium pyrophosphate for 15 min; (ii) the sonication conditions, where the sediments were sonicated for 3 min in an ice or water bath, with interruption and 30-s manual shaking every minute; (iii) the extraction efficiency between the dilution and centrifugation methods, where the sediments were diluted or centrifuged for 800 *g* × 1 min, and the recovery efficiency of two subsequent washing steps after centrifugation was also tested; and (iv) −80°C preservation and slide preservation methods, where fresh sediment samples were processed and counted within 2 h after collection, and the slides were then stored at −20°C and recounted after 30 and 90 days of storage, while the other sediment subsamples were snap-frozen in liquid nitrogen, stored at −80°C for 7, 30, 60, and 90 days, and then processed and counted. All these tests were performed using the sandy and muddy-sand sediments from the intertidal zone of Qingdao Bay, except the sonication assays, which were only performed using the sandy sediment samples. Each test was performed with three replicates.

### Enumeration of Viruses and Prokaryotes

The VA and PA were determined using an epifluorescence microscope (Zeiss Axioplan) following the optimized procedure of Wei and Xu (2017). Briefly, after adding 0.3 mL of a 50 mmol/L sodium pyrophosphate solution (3 mmol/L final concentration), the subsamples were gently shaken and incubated for 15 min in the dark and then sonicated in a water bath for 3 min (with 30 s intervals every minute). The slurries were then diluted 1,000–10,000-fold and 0.5-mL were filtered through Anodisc aluminum oxide filters (0.02-μm pore size). The filters were stained with 20 μL of SYBR Green I (diluted 20-fold in 0.02-μm filtered MilliQ water) for 20 min in the dark, rinsed on the back of the filters with 0.5 mL of 0.02-μm filtered MilliQ water, and then mounted to a microscopic slide using 25 μL of anti-fade solution [PBS (0.05 mol/L Na_2_HPO_4_ and 0.85% (wt/vol) NaCl):glycerol (1:1) and 0.1% *p*-phenylenediamine]. The slides were viewed under a blue light. Each filter was viewed for 40 fields or at least 400 particles for the enumeration of viruses and prokaryotes. The disposable supplies were pre-sterilized, the reagents and solutions were freshly prepared with 0.02-μm filtered MilliQ water. The parallel blank was run to avoid virus and prokaryotic particles pollution.

### Meta-Data Collection, Filtering and Processing

Articles were primarily obtained from the references of published reviews and datasets (Danovaro et al., 2008b; Jacquet and Parikka, 2016; Parikka et al., 2016) and were also gathered using online search engines and databases with the keywords ‘virus and sediment,’ etc. Articles using transmission electron microscopy (TEM) were excluded. Data using the viral and prokaryotic abundances of pore water to represent that of sediments were excluded, because this method has been proved to significantly underestimate the true abundances (Helton et al., 2006). Only data of the surface sediments (mostly 0–1 cm, with a few data points from 0–2 and 0–3 cm samples) were collected for analysis. The selected articles should provide data for at least two out of three assayed parameters (VA, PA, and VPR), and the missing values (if any) were calculated by the given ones. For articles that use graphs to show data instead of giving specific values, the data were estimated from the graphs. Because data from each studied site were considered as an independent sample for all analyses, more than one site was obtained from some studies. For seasonal data of individual sites, only the mean value was used for the analysis. The recorded sites were categorized into different ecosystems according to their habitats (e.g., marine, brackish, freshwater, hydrothermal vents, and cold seeps) and water depth (for marine data only), and the primary processing protocols are also listed in the Supplementary Information (Supplementary Table S1).

### Statistical Analysis

Statistical analysis was performed using SPSS 16.0, and the Student *t*-test, the Mann–Whitney *U* test and the Kruskal–Wallis *H* test were used to compare the VPR values under different conditions. Spearman’s rank correlation coefficients were used to analyze the relationships between environmental factors and VA, PA, and VPR. The package BIOENV of PRIMER 6 was used to analyze the relationships between the VA, PA, and VPR and environmental factors.

## Results

### Impact of Processing Methods on VPR

**FIGURE 2:**
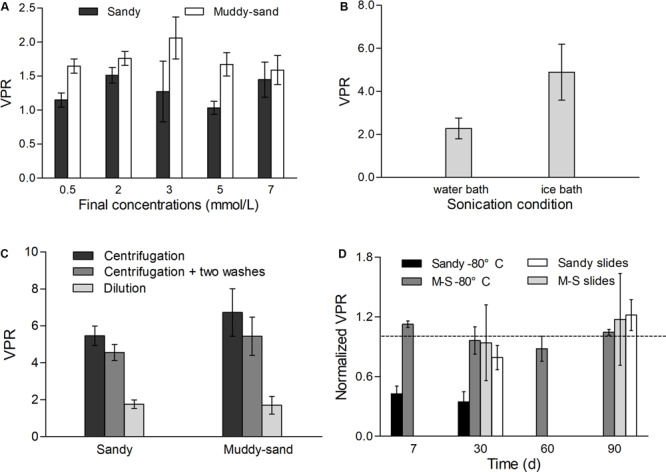
The impact of processing methods on VPR, each test was performed with three replicates. Panel **(A)** shows the effect of final concentration of sodium pyrophosphate in sandy and muddy-sand sediments. Panel **(B)** shows the effect of sonicating in the water bath and ice bath in sandy sediment. Panel **(C)** shows the effect of dilution, centrifugation, and centrifugation with two washes on VPR in sandy and muddy-sand sediments. And panel **(D)** shows the effects of –80°C preservation for sandy and muddy-sand (M-S) sediments and slides preservation for sandy slides and muddy-sand (M-S) sediments on VPR. VPR values were normalized to the VPR of fresh sediments (dotted line).

The evaluation and optimization of processing methods for VA and PA determinations were essentially described in a Chinese article (Wei and Xu, 2017). In this study, we focused on the impact of sample processing methods on VPR. The optimal final concentration of sodium pyrophosphate for the extraction of both viruses and prokaryotes was 3 mmol/L, indicating that the VPR values determined at this concentration are the most accurate (Wei and Xu, 2017), though the Kruskal–Wallis *H* test showed no difference in VPR values determined for different final concentrations from both sandy and muddy-sand sediments (Figure 2A). The test of sonication conditions showed a higher loss of PA versus VA (72.4 and 37.2%, respectively; Wei and Xu, 2017) when adding ice during sonication, resulting in an approximately 2.2-fold overestimation of VPR (Figure 2B), while the difference was not significant (The Student *t*-test, *p* > 0.05).

Compared to the dilution method, centrifugation caused a much higher loss of PA than VA in both sandy and muddy-sand sediments, with approximately 40–50% of VA and 80–90% of PA being lost (Wei and Xu, 2017). Thus, the VPR values were overestimated by 3.1- and 4.0-fold in the two types of sediments, respectively (Figure 2C). After the additional two washing steps, which are typically added to reduce the loss caused by centrifugation in many studies, only approximately 60–70% of the total viruses and merely 20–30% of the total prokaryotes were retrieved from the two types of sediments. Therefore, even when adding an additional washing step, the VPR was still overestimated by 2.7- and 3.2-fold.

Virus-to-prokaryote ratio values didn’t change significantly in both the sandy and muddy-sand sediments over 3 months using the slide preservation (Kruskal–Wallis H test, *p* > 0.05) (Figure 2D). In contrast, the −80°C preservation showed varied efficiency for the two types of sediments. The rate of decrease in viruses was significantly higher than that of prokaryotes in the sandy sediment, leading to a continuous decrease in VPR. After 1 week of preservation, the VPR was reduced by nearly 60%, and dropping to 34.7% of the initial value after 30 days. For the muddy-sand samples, the VPR showed no significantly change over 90 days of −80°C preservation (Kruskal–Wallis H test, *p* > 0.05).

### Temporal Distributions of VA, PA, and VPR With Respect to Environmental Factors

**FIGURE 3:**
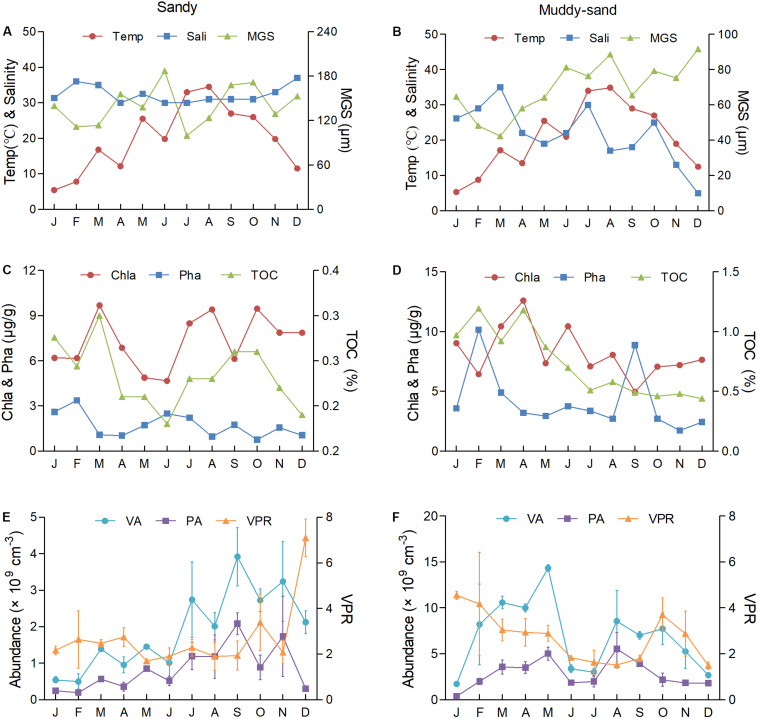
The monthly variation of VA, PA, VPR, and environment factors at the sandy site S **(A,C,E)** and the muddy-sand site M-S **(B,D,F)** in the intertidal zone of Qingdao Bay. Temp, the temperature of the surface sediment; MGS, median grain size; Chla, chlorophyll *a*; Pha, pheophytin *a*; TOC, total organic carbon.

The environmental conditions at the two investigated sites were significantly different (Table 1). The temperature of the surface sediment was the lowest in January and highest in August. At the sandy site S, the salinity was stable throughout the year (mean ± SD, 32.3 ± 2.32) and the MGS fluctuated slightly (140.9 ± 25.69 μm) (Figure 3A). The Chla (7.3 ± 1.68 μg/g) peaked in March and then rapidly fell to the lowest value in June before rebounding. The Pha (1.7 ± 0.77 μg/g) showed an opposite trend to that of Chla, while the TOC (0.28 ± 0.03%) varied similar to Chla (Figure 3C). At the muddy-sand site M-S, the salinity was much lower and greatly fluctuated (21.77 ± 7.79) than that of site S, and the MGS was also lower and showed an increasing trend (69.66 ± 14.59 μm) (Figure 3B). The Chla (8.20 ± 2.01 μg/g), Pha (4.20 ± 2.50 μg/g), and TOC (0.73 ± 0.27%) were much higher than those observed at site S, all showing a decreasing trend during the investigated period (Figure 3D).

**TABLE 1:** Comparison and Spearman correlations of viral abundance (VA), prokaryotic abundance (PA), and virus-to-prokaryote ratio (VPR) and environmental factors at the sandy site S and the muddy-sand site M-S.

	**Sites**	**VA**	**PA**	**VPR**	**Chla**	**Pha**	**TOC**	**MGS**	**Temperature**	**Salinity**	**Water content**
S vs. M-S^a^		< 0.000**	< 0.000**	0.887	0.319	< 0.000**	< 0.000**	< 0.000**	0.799	< 0.000**	< 0.000**
VA	S	**−**	< 0.000**	0.729(−)	0.379	0.265(−)	0.991(−)	0.812	0.013*	0.742(−)	0.342
	M-S	−	0.001**	0.527	0.762	0.762	0.208	0.075(−)	0.527	0.712	0.331
PA	S	−	−	0.167(−)	0.443	0.354(−)	0.778	0.966(−)	< 0.000**	0.279(−)	0.602
	M-S	−	−	0.697(−)	0.762(−)	0.697	0.633	0.471(−)	0.042*	0.966(−)	0.513
VPR	S	−	−	−	0.175	0.404(−)	0.687	0.897	0.124(−)	0.423	0.145
	M-S	−	−	−	0.914	0.191	0.036*	0.011*(−)	0.063(−)	0.037*	0.226

^*a*^The Mann–Whitney U test of microorganisms’ parameters and environmental factors at sites S and M-S; Bold*, significant (p < 0.05); Bold**, strongly significant (p < 0.01); (−), negative correlation; Chla, chlorophyll a (μg/g); Pha, phaeophytin a (μg/g); TOC, total organic carbon (%); MGS, median grain size (μm).

The VA and PA showed a similar monthly trend at site S, where both began to increase in February, peaked in September and then decreased (Figure 3E). The VA at site S was (1.89 ± 1.05) × 10^9^cm^–3^ (5.0 × 10^8^ cm^–3^–3.92 × 10^9^ cm^–3^) and the PA was (8.44 ± 5.82) × 10^8^ cm^–3^ (1.92 × 10^8^ cm^–3^–2.08 × 10^9^ cm^–3^). The VA and PA at site M-S were much higher (*p* < 0.01, Table 1) and showed a similar monthly trend as those observed at site S (Figure 3F), both increasing from January to May, followed by a rapid decrease in June and July before rebounding in August and then decreasing again. The VA at site M-S was (6.88 ± 3.63) × 10^9^cm^–3^ (1.73 × 10^9^ cm^–3^–1.43 × 10^10^ cm^–3^), and the PA was (2.80 ± 1.45) × 10^9^ cm^–3^ (3.80 × 10^8^ cm^–3^–5.54 × 10^9^ cm^–3^) (Supplementary Table S1).

The VPR values were relatively similar, with values of 2.69 ± 1.40 (1.71–7.10) at site S and 2.70 ± 1.02 (1.52–4.55) at site M-S (*p* > 0.05, Table 1). The VPR values at site S were relatively steady from January to April (winter to early spring), decreasing and maintaining the lowest values from May to September (late spring to early autumn), and then increasing with fluctuations from October to December before peaking in December. Overall, the VPR values at site S were higher in the winter and spring and lower in the summer, while the difference was not significant. The annual VPR variation at site M-S was similar to that observed at site S, with higher VPR values observed in the winter and lower values measured in the spring and autumn, with the lowest value detected in summer.

The Pha and TOC at site M-S were 2.4- and 2.6-fold higher than those at site S, respectively. Similarly, the VA and PA at site M-S were 3.6- and 3.3-fold higher than those observed at site S, respectively. In contrast, the VPR of the two sites were rather similar. Spearman’s correlation analysis showed that the VA at site S and the PA at both sites was positively correlated with the temperature (Table 1). No significant correlation was observed between VPR and any environmental factor at site S, while VPR was positively correlated with the TOC and salinity and was negatively correlated with the MGS at site M-S. At both sites, the VA and PA were positively correlated with each other, while there was no significant correlation between the VPR and either the VA or PA.

### Spatial Distributions of VA, PA, and VPR From the Intertidal Zone to the Deep-Sea Plains

The Chla, MGS, and temperature showed a downward trend from the intertidal zone to the deep-sea plain, while the salinity and water content of the sediments showed an opposite trend (Table 2). The Pha was highest in the continental shelves, followed by the intertidal zone, and lowest in the deep-sea plains. The TOC was similar in all areas. Only Chla and water depth were significantly different among the environmental factors at the intertidal zone and continental shelf sites (Table 3). Most of the measured environmental factors in the shallow-sea areas (intertidal zone and continental shelves), the Northwest Pacific Ocean and the Philippine Basin were significantly different from one another (Table 3).

**TABLE 2:** Mean values of VA, PA, and VPR and environmental factors in the studied sea areas.

**Parameters**	**Intertidal zone**	**Yellow Sea**	**East China Sea**	**Northwest Pacific**	**Philippine Basin**
VA (×10^9^cm^–3^)	4.383.66	4.151.55	4.431.37	1.290.38	0.0850.038
PA (×10^9^ cm^–3^)	1.821.47	1.760.75	1.890.72	0.980.24	0.170.078
VPR	2.701.22	2.891.21	2.520.83	1.380.38	0.500.057
Chla (μg/g)	7.761.90	0.300.24	1.420.60	0.120.069	undetectable
Pha (μg/g)	2.962.23	5.651.14	5.102.22	1.750.61	0.130.064
TOC (%)	0.510.30	0.760.27	0.570.14	0.630.10	0.490.25
Temperature (°C)	20.309.18	9.821.21	23.122.37	1.04^a^	1.680.079
Salinity	27.047.80	32.400.50	33.312.64	34.69^a^	34.680.0082
MGS (μm)	105.2841.29	14.1313.79	45.6660.89	11.202.15	4.600.98
Water depth (m)	0	63.6714.41	55.6714.35	5811.0118.6	5308.0399.79
Water content (%)	0.350.06	0.530.07	0.480.12	0.650.05	0.660.06
Number of sites analyzed	2^b^	9	9	7	4

**Chla, chlorophyll a; Pha, phaeophytin a; TOC, total organic carbon; MGS, median grain size; ^*a*^represented by data of one site at North Pacific (160°E, 35°N, water depth 4,330 m); ^*b*^data calculated with the annual mean at each site. –, not determined.**

**TABLE 3:** The Mann–Whitney *U* test of the benthic VA, PA, and VPR and environmental factors in different sea areas.

**Sites**	**VA**	**PA**	**VPR**	**Chla**	**Pha**	**TOC**	**Temperature**	**Salinity**	**MGS**	**Water depth**	**Water content**	***N***
QBI vs. YS	0.909	0.909	0.909	0.036*	0.073	0.582	0.036*	0.036*	0.036*	0.036*	0.036*	11
QBI vs. ECS	1.000	0.909	0.909	0.036*	0.327	0.909	0.218	0.073	0.218	0.036*	0.327	11
YS vs. ECS	0.546	0.489	0.489	< 0.000**	0.605	0.340	< 0.000**	0.004**	0.340	0.136	0.340	18
Conti vs. QBI	0.947	0.853	1.000	0.011*	0.126	0.674	0.758	0.211	0.063	0.011*	0.095	20
Shallow vs. NP	< 0.000**	0.005**	0.001**	0.001**	< 0.000**	1.000	−	−	0.850	< 0.000**	< 0.000**	27
Shallow vs. PB	< 0.000**	< 0.000**	< 0.000**	< 0.000**	< 0.000**	0.210	< 0.000**	< 0.000**	0.001**	< 0.000**	0.002**	24
PB vs. NP	0.006**	0.006**	0.006**	0.006**	0.006**	0.230	−	−	0.006**	0.109	1.000	11
Shallow vs. Deep	< 0.000**	< 0.000**	< 0.000**	< 0.000**	< 0.000**	0.328	< 0.000**	< 0.000**	0.155	< 0.000**	< 0.000**	31

Bold*, significant (p < 0.05); Bold**, exact significant (p < 0.01); N, the total number of studied sites in the compared habitats; QBI, the intertidal zone of the Qingdao Bay, data calculated with the annual mean at each site; YS, the south Yellow Sea; ECS, the East China Sea; NP, the Northwest Pacific; PB, the Philippine Basin; Conti, continental shelves, including all sites of the YS and the ECS; Shallow, shallow-sea areas, including all sites of the QBI, YS, and ECS; Deep, deep-sea areas, including all sites of PB and NP; Chla, chlorophyll a (μg/g); Pha, phaeophytin a (μg/g); TOC, total organic carbon (%); MGS, median grain size (μm).

The VA and PA varied from 10^7^ to 10^9^ cm^–3^ in all the investigated areas, with the highest values observed in the East China Sea and those in the Philippine Basin being the lowest (Table 2). There was no significant difference for both the VA and PA between the intertidal zone and continental shelves (Table 3). In the shallow-sea areas, the VA was (4.30 ± 1.64) × 10^9^ cm^–3^ (1.89 × 10^9^–6.88 × 10^9^ cm^–3^) and the PA was (1.83 ± 0.79) × 10^9^ cm^–3^ (8.44 × 10^8^–3.53 × 10^9^ cm^–3^). In the Northwest Pacific Ocean, the VA was one-third of that observed in the shallow-sea areas (8.10 × 10^8^–1.77 × 10^9^ cm^–3^), while the PA was approximately one-half of that observed in the shallow-sea areas (6.3 × 10^8^–1.31 × 10^9^cm^–3^). The VA in the Philippine Basin was more than one order of magnitude lower than that observed in the Northwest Pacific Ocean (3.22 × 10^7^–1.39 × 10^8^ cm^–3^), while the PA was one order of magnitude lower than that observed in the shallow-sea areas and less than one-fifth of that detected in the Northwest Pacific Ocean (6.46 × 10^7^ to 2.84 × 10^8^ cm^–3^) (Supplementary Table S1).

The VPR ranged from 0.43 to 4.81 in all investigated areas. Similar to the VA and PA, there was no significant difference in VPR values between the intertidal zone and the continental shelves (Table 3). The VPR was 2.71 ± 1.02 (1.16–4.81) in the shallow-sea areas, and the mean value was slightly higher in the south Yellow Sea than that observed in the intertidal zone and the East China Sea. The mean VPR value in the Northwest Pacific Ocean was approximately one-half of that observed in the shallow-sea areas (0.71–2.05), while the VPR in the Philippine Basin was only one-fifth of that observed in the shallow-sea areas, ranging from 0.43 to 0.59 (Table 2 and Supplementary Table S1).

**FIGURE 4:**
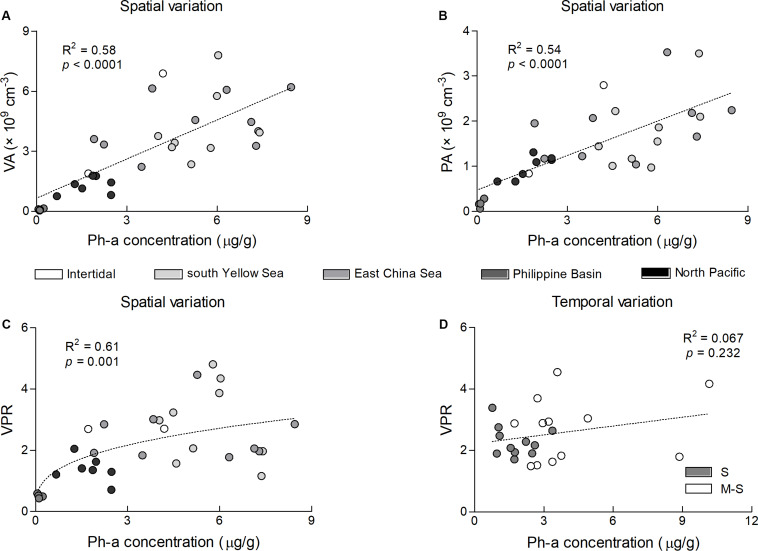
Spatial and temporal patterns of VA, PA, and VPR along with the sediment Pha gradient. Panel **(A)** shows the spatial pattern of VA, *y* = 0.6508*x* + 0.6513, **(B)** shows the spatial pattern of PA, *y* = 0.2546*x* + 0.4741 and **(C)** shows the spatial pattern of VPR, *y* = 1.2897*x*^0.3837^. Panel **(D)** shows the temporal pattern of VPR in the intertidal zone, where there was no correlation between VPR and the Pha.

The BIOEVN analysis showed that the most relevant environmental factors impacting the mesoscale spatial pattern of VPR were the combination of the sediment Pha content and water depth, and the water depth and Pha content were the most relevant to the VA and PA spatial patterns, respectively (Table 4). Regression analysis showed that the VA, PA, and VPR values had a much higher goodness of fit with the Pha content than with the water depth (Figure 4 and Supplementary Figure S1). From the intertidal zone to the deep-sea plains, the VA and PA values both exhibited a good linear correlation with the Pha content, while the exponential relationship rather than the linear correlation (*R*^2^ < 0.5) better explained the spatial pattern of the VPR values along with the Pha content (Figures 4A–C).

**TABLE 4:** The BIOENV analysis of the studied areas.

	**Shallow-sea areas**		**Deep-sea areas**		**All areas**	
	**Corr.**	**Combinations**	**Corr.**	**Combinations**	**Corr.**	**Combinations**
Virus	0.350	Chla, TOC, Salinity	0.744	T	0.609	Depth
	0.348	Chla, TOC, Depth	0.711	Pha, T, MGS, Depth	0.593	Pha, Depth
	0.347	Chla, TOC, T, Depth	0.684	Pha, T	0.551	Pha, T, Depth
Prokaryote	No correlation *p* = 0.198		0.796	T	0.395	Pha
			0.795	Pha, TOC, T, MGS, Depth	0.394	Pha, Depth
			0.773	Chla, TOC, T, MGS, Depth	0.355	Pha, T
VPR	No correlation *p* = 0.974		0.561	T, Depth	0.386	Pha, Depth
			0.558	Chla, T, Salinity, MGS, Depth	0.370	Pha, T, Depth
			0.548	Chla, T, MGS, Depth	0.354	T, Depth

**Corr., correlation coefficients; the top 3 combinations are given. Chla, chlorophyll-a (μg/g); Pha, phaeophytin-a (μg/g); TOC, total organic carbon (%); T, the temperature of the surface sediments in intertidal zone and the bottom water temperature on the continental shelves and deep-sea plains (°C); MGS, median grain size (μm); WC, water content (%). The temperature and salinity of North Pacific was represented by the data of one site (160°E, 35°N, water depth 4,330 m).**

### Meta-Analysis of VPR Data

A total of 135 data points from the surface sediments of various habitats were collected and compiled in Supplementary Table S1, among which 115 were obtained from marine habitats, from the intertidal zone to the abyss. The marine benthic VPR ranged from 0.11 to 98.0, with an average of 6.33 ± 15.64. The other 20 data points were collected from freshwater habitats, where the VPR ranged from 0.13 to 23.42, with an average of 3.58 ± 4.71. Most marine benthic VPR data were obtained using the dilution method, and only one-quarter of the marine benthic data were obtained using the centrifugation method (with and without subsequent washes) (Table 5).

**TABLE 5:** Viral abundance, PA, and VPR values in different benthic habitats under centrifugation and dilution treatments (data from various publications listed in Supplementary Table S1).

**Treatments**	**Habitat types**	**VA (×10^8^)^*a,b*^**	**PA (×10^8^)^*a,b*^**	**Unit**	***N***	**VPR^*a*^**	***N***
**Centrifugation**	Intertidal zone	13.25 (2.0–24.50)	0.23 (0.20–0.25)	cm^–3^	2	54.00 (10.00–98.00)	2
	Continental shelf	92.53 ± 158.77 (0.27–429.90)	1.54 ± 2.21 (0.09–7.76)	cm^–3^	12	27.02 ± 33.83 (2.87–96.73)	12
	Mesobenthic zone	24.50	2.25	cm^–3^	1	10.89	1
	Bathyal zone	9.90	0.59	cm^–3^	1	9.11 ± 5.47 (4.51–16.80)	3
		6.83 (5.46–8.20)	1.29 (0.08–1.29)	g^–1^	2		
	Brackish water	5.11 ± 1.48 (2.76–7.50)	1.70 ± 1.07 (0.32–3.80)	cm^–3^	7	4.11 ± 2.09 (1.37–8.63)	7
	Freshwater	32.01 ± 17.22 (17.33–63.64)	4.98 ± 3.56 (0.74–10.50)	cm^–3^	5	9.01 ± 6.73 (2.71–23.42)	6
		350.00	129.00	g^–1^	1		
	Cold seep	3.61 ± 3.55 (0.05–8.80)	0.75 ± 0.77 (0.14–2.18)	cm^–3^	5	17.51 ± 18.42 (0.36–66.36)	12
		56.08 ± 50.89 (6.08–130.70)	2.49 ± 2.09 (0.22–6.27)	g^–1^	7		
**Dilution**	Intertidal zone	41.90 ± 41.41 (0.69–83.50)	14.58 ± 12.15 (0.93–27.97)	cm^–3^	3	2.52 ± 0.94 (0.74–3.45)	3
		**43.82 (18.86**–**68.79)**	**18.21 (8.44**–**27.97)**	cm^–3^	**2**	**2.70 (2.69**–**2.70)**	**2**
	Continental shelf	7.08 ± 6.70 (1.20–24.96)	8.29 ± 8.23 (1.43–28.78)	cm^–3^	11	1.88 ± 1.97 (0.19–11.00)	32
		32.52 ± 46.50 (2.00–220.00)	13.61 ± 8.31 (2.63–35.00)	g^–1^	21		
		**42.88 ± 14.70 (22.20**–**77.92)**	**18.27 ± 7.38 (9.70**–**35.30)**	cm^–3^	**18**	**2.69 ± 1.05 (1.16**–**4.81)**	**18**
	Mesobenthic zone	9.75 ± 4.51 (3.00–17.60)	4.58 ± 2.24 (1.80–8.20)	g^–1^	7	1.85 ± 0.61 (1.00–2.87)	7
	Bathyal zone	9.90	1.70	cm^–3^	1	2.09 ± 2.06 (0.11–6.10)	19
		5.31 ± 7.19 (0.36–23.75)	3.73 ± 2.72 (0.16–11.00)	g^–1^	18		
	Abyssal benthic zone	14.01 ± 5.53 (0.86–19.01)	8.96 ± 5.66 (1.84–16.81)	g^–1^	8	1.81 ± 1.11 (0.47–4.08)	8
		**8.50 ± 6.54 (0.33**–**17.70)**	**6.87 ± 4.38 (0.65**–**13.10)**	cm^–3^	**11**	**1.04 ± 0.51 (0.43**–**2.04)**	**11**
	Brackish water	8.00	8.46	g^–1^	1	0.95	1
	Freshwater	19.52 ± 24.74 (0.11–63.37)	13.01 ± 17.14 (0.62–55.17)	cm^–3^	13	2.20 ± 2.48 (0.13–9.10)	14
		11.70	9.55	g^–1^	1		
	Hydrothermal vents	14.18 ± 8.78 (3.69–31.87)	5.26 ± 3.31 (1.16–11.31)	g^–1^	8	3.52 ± 2.51 (1.06–7.90)	8

**^*a*^Mean ± SD (minimum–maximum); ^*b*^VA and PA were expressed as cm^–3^ (including ml^–1^) and g^–1^; Bold: data of the present study; N, number of sites.* Data from Danovaro and Serresi (2000), Danovaro et al. (2002, 2005, 2008a, 2009), Farnell-Jackson and Ward (2003), Hewson and Fuhrman (2003), Middelboe et al. (2003, 2006), Brussaard (2004), Glud and Middelboe (2004), Mei and Danovaro (2004), Bettarel et al. (2006), Duhamel and Jacquet (2006), Filippini et al. (2006), Fischer et al. (2006); Corinaldesi et al., 2007, 2010, 2012, Filippini and Middelboe (2007), Seymour et al. (2007), Manini et al. (2008), Patten et al. (2008), Siem-Jørgensen et al. (2008), Wieltschnig et al. (2008); Kellogg, 2010, Helton et al. (2011), Borrel et al. (2012), Carreira et al. (2013), Luna et al. (2013), Wróbel et al. (2013), Rastelli et al. (2016), He et al. (2017), Vandieken et al. (2017).*

**FIGURE 5:**
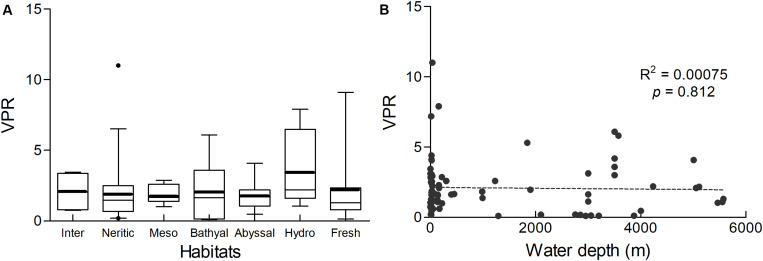
**(A)** The VPR values obtained by dilution from different benthic habitats. Data were shown in Supplementary Table S1. The boxed region shows the 25th and 75th percentiles and the whiskers show the 5–95%. The thin line indicates the median and the bold line indicates the mean. Outliers are shown as black points. From left to right, the box plots represent the VPR values of the intertidal zone, continental shelf, mesobenthic zone, bathyal zone, abyssal benthic zone, hydrothermal vents and freshwater sediments, respectively. **(B)** The spatial pattern of VPR values along with the water depth in marine sediments.

In the corresponding habitats, each of the mean VPR values obtained by dilution was much lower than that obtained by centrifugation. Overall, the average benthic VPR obtained by the dilution method was only 1.96 ± 1.77, while that obtained by centrifugation was approximately 9.1-fold higher (17.77 ± 25.72). Moreover, nearly 60% of the VPR obtained by the dilution method fell into the range of 0 to 2, with more than 90% varying between 0 and 5, and only one value exceeded 10 (Figure 5A). The mean VPR values fluctuated within a narrow range of variation from 0.95 to 3.52 in all benthic habitats. In contrast, the mean VPR values obtained using the centrifugation method were highly variable (4.11–54.0), where approximately 35% of the VPR values fell into the range of 0 to 5, with more than 50% varying between 5 and 50 and more than 10% being higher than 50 (Table 5 and Supplementary Table S1).

In marine benthic habitats, the maximum and minimum VPR were obtained from hydrothermal vents and the abyss, respectively. The marine benthic VPR showed a slight downward trend from the intertidal zone to the abyssal plain, but no significant correlation was observed between the VPR and water depth (Figure 5B). The mean VPR of freshwater sediments was slightly lower than that observed for the intertidal zone. There was no significant difference in the VPR in different benthic habitats, including hydrothermal vents and freshwater sediments (Kruskal–Wallis *H* test, *p* > 0.05). The VPR in marine habitats was significantly correlated with VA (*p* < 0.01), while there was no correlation with PA. VA and PA were significantly correlated with each other (*p* < 0.01). Both VPR and VA had no correlation with water depth, while PA was significantly negatively correlated with the water depth (*p* < 0.01). In freshwater habitats, there was no correlation was observed between the VPR and VA, PA and water depth, except for the positive correlation observed between VA and PA.

## Discussion

### Impact of Extraction and Storage Procedures on the VPR

The results of the methodological evaluation and meta-analysis of the VPR data support our hypothesis that the VPR in surface sediments has been overestimated due to the significantly lower extraction efficiency of prokaryotes than viruses caused by sample processing. In particular, centrifugation had a significant influence on the VA, PA, and VPR values, as indicated in previous studies (Glud and Middelboe, 2004; Siem-Jørgensen et al., 2008; Danovaro and Middelboe, 2010). Our results showed that several subsequent washes could not compensate for the decrease in VA and PA caused by centrifugation. This result is consistent with Siem-Jørgensen et al. (2008) but opposite to that reported in some previous studies (Danovaro et al., 2001; Hewson and Fuhrman, 2003; Middelboe et al., 2003; Vandieken et al., 2017). Moreover, our results indicate that the finer the sediments treated, the greater the overestimation of the VPR. The meta-analysis showed a higher overestimation than the laboratory test results, because most marine sediment particles were finer than the intertidal sediments used in the present study. Different centrifugation conditions may also influence the results. For instance, the maximum VPR value determined in the present study was obtained by using the recorded maximum centrifugal force and time (Helton et al., 2011).

Other sample extraction procedures have limited impact on VPR. The ice-bath sonification step could lead to an overestimation of VPR, but its use was limited in published articles (Supplementary Table S1). The slide and −80°C preservation methods for muddy-sand sediments could effectively maintain the VPR value, while the −80°C preservation method was less efficient for sandy sediments, which should be noted in subsequent research.

### Temporal and Spatial Distribution of Benthic VPR Values

Our results showed that the benthic VPR is much lower and less varied across temporal and spatial scales than the prevailing view. The VPR values obtained in the present study fall within the range of those obtained by the dilution method in previous studies, fluctuating much less than the range when the results obtained using the centrifugation method were included in the meta-data analysis (0.001–225; Parikka et al., 2016). During the annual survey of the intertidal sediments, the environmental factors between the two investigated sites differed greatly, which was clearly reflected in the viral and prokaryotic abundance at the two sites. Large fluctuations in VA and PA values were observed at both sites, exceeding an order of magnitude, as observed in previous studies (Fischer et al., 2003; Helton et al., 2011). Nonetheless, the VPR values at both sites were rather similar and less varied. Similar variations in VPR values were observed in studies by Fischer et al. (2003), Filippini et al. (2006), and Siem-Jørgensen et al. (2008), in which the VPR values fluctuated within a very narrow range (0.9–3.2, 0.9–1.9, and 1.0–2.8, respectively). In contrast, Helton et al. (2011) observed extremely high interannual dynamics of VPR values, which was likely caused by the use of the centrifugation method.

We observed a modest trend in the seasonal variation of VPR values, which were lower at high temperature at both sites. Fischer et al. (2003) and Filippini et al. (2006) also obtained the lowest VPR values in the summer. Previous studies indicated that higher temperature and stronger solar radiation in warmer months may accelerate viral decay (Yates et al., 1987; Fischer et al., 2003; Danovaro et al., 2008b). In contrast, both VA and PA at site S showed significant seasonal patterns related to the temperature of surface sediment. Such a correlation was also observed at site M-S, where sharp decreases in VA and PA values occurred in June and July, which is likely relevant to the outbreak and accumulation of the green macroalga *Ulva prolifera* from July to August. The degradation of *Ulva prolifera* could release a large amount of toxic sulfide (Norkko and Bonsdorff, 1996; Wu et al., 2010), leading to acidification in the sediments (Zhang et al., 2019), thereby increasing the viral and prokaryotic decay. The VPR values at the two sites did not correlate with either the VA or PA, as observed in all other temporal distribution studies in benthic habitats (Fischer et al., 2003; Baker and Leff, 2004; Filippini et al., 2006; Jackson and Jackson, 2008; Siem-Jørgensen et al., 2008; Helton et al., 2011), indicating the close numerical dependence of viruses on their hosts and the stability of the virus–host relationship in intertidal benthic habitats (Hara et al., 1991; Wommack and Colwell, 2000).

The VPR is generally considered to decrease with water depth, but this view is not supported by the results of the present study, since there was no significant correlation between the VPR and water depth. Our study indicates a spatial pattern of decrease in VPR, VA, and PA values along the trophic gradients, consistent with the results of several previous studies (Danovaro and Serresi, 2000; Hewson et al., 2001; Danovaro et al., 2002; Parikka et al., 2016). Our results showed that the VPR was positively correlated with the sediment Pha content, which represents the cumulative amount of chlorophyll *a* settled from the upper water column to the bottom and can serve as an indication of the environmental trophic status (Boyer et al., 2009). The exponential relationship between the VPR value and Pha content showed that the VPR was more spatially sensitive to the changes in the Pha content in nutritionally inadequate environments (Figure 4C), indicating that nutritional conditions likely play a more important role than water depth in regulating the spatial distribution of the benthic VPR. Based on the amount of particulate organic carbon (POC) deposited from the upper water column to the bottom, our investigated areas could be divided into three nutrient levels. The shallow-sea areas, including the south Yellow Sea (approximately 15 g Corgm^–2^yr^–1^; Hu et al., 2016) and East China Sea (approximately 19 g Corgm^–2^yr^–1^; Jiao et al., 2018) were the most nutrient rich, followed by the Northwest Pacific Ocean (2–3 Corgm^–2^yr^–1^; Watling et al., 2013), and the Philippine Basin was the most barren (0–1 g Corgm^–2^yr^–1^; Watling et al., 2013). The VPR values were significantly lower in the oligotrophic deep-sea areas, including the Northwest Pacific Ocean and Philippine Basin, than in the shallow-sea areas, and they were much higher in the Northwest Pacific Ocean than in the Philippine Basin. In contrast, the VPR, VA, and PA values were rather similar and slightly varied, regardless of the considerable differences in the environmental conditions in the nutrient-rich shallow-sea areas. On the other hand, marine particles are prone to concentrate viruses and transfer them from water column to sediments (Dang and Lovell, 2015), which may also affect the distribution of benthic VPR. The impact of such transmission on sediment viruses requires further research.

### Different VPR Values Between Pelagic and Benthic Habitats

Our results showed that the mean VPR value in sediments is approximately one order of magnitude lower than that observed in the pelagic systems (1.96 vs. 21.9; Parikka et al., 2016; present study), suggesting a much lower predominance of viruses over hosts in benthic versus pelagic habitats. The primary reason for the low benthic VPR value is that viruses are relatively less abundant compared to the relatively higher PA observed in sediments than in water columns. The PA in benthic habitats is approximately three orders of magnitude higher than that observed in pelagic habitats (10^8^–10^9^ g^–1^ vs. 10^5^–10^6^ ml^–1^), while the VA is only approximately two orders of magnitude higher in benthic than that observed in pelagic habitats (10^7^–10^10^ g^–1^ vs. 10^6^–10^8^ ml^–1^) (Suttle, 2005; Danovaro et al., 2008b; Meng et al., 2011). Previous studies showed that the virus burst size between sediments and water columns was rather similar (11–106 viruses cell^–1^ vs. 10–100 viruses cell^–1^; Danovaro et al., 2008a, b), indicating a much lower frequency of lytic infection in sediments than in water columns (Danovaro et al., 2002). The BS value of some sediment viruses is very high, as in the case of benthic phage S0112 (BS = 1170, Wang et al., 2019), in which case the proportion of lytic infection will be even lower. This result is well demonstrated by the significantly low number of visibly infected cells in marsh benthic habitats, where Filippini et al. (2006) detected viruses in only 4 of approximately 15,000 bacterial cells, while nearly 300 of approximately 5,000 cells were visibly infected in the upper waters. Our finding of universally low VPR values in benthic habitats indicates that it is common for only a small proportion of the prokaryotes to be affected by lytic viruses in sediments. The fluctuation in benthic VPR values was also much lower than that observed in pelagic habitats on both temporal and spatial scales (0.008–2150; Parikka et al., 2016). Considering that the VA and PA values were significantly correlated with each other (*p* < 0.01), the less varied benthic VPR is likely to be primarily attributed to the low lytic infection rates. This phenomenon likely partly explains the much lower dynamics of virus–host interactions in benthic habitats than in pelagic habitats.

Virus–host encounters increase with host cell density (Wiggins and Alexander, 1985), making benthic habitats with a high density of host cells favorable for virus proliferation (Danovaro et al., 2008b). However, the characteristics of benthic microhabitats also hinder the spread of viruses. The transport of viruses dominated by Brownian diffusion can be limited by the high tortuosity of the sediment (Drake et al., 1998; Fischer et al., 2003). In addition, the viral decay in sediments is likely to be high, as the viral adsorption to sediment particles is responsible for most of the viral decay or the loss of infectivity (Suttle and Chen, 1992; Fischer et al., 2003). In addition, the characteristics of benthic hosts are unfavorable for viral proliferation. The much higher diversity of both prokaryotes and viruses than those in water columns increases the difficulty for viruses to find suitable hosts in sediments, as the viruses are host-specific (Fischer et al., 2003; Filippini et al., 2006; Danovaro et al., 2008b). Luna et al. (2002) observed that approximately 96% of prokaryotic cells were dead or dormant in coastal marine sediments, making it difficult for viruses to encounter active hosts and prolonging the viral latent period duration (Wiggins and Alexander, 1985). These disadvantages may benefit other viral lifestyles, including lysogenic and chronic infections, which result in the release of fewer viral particles and are hard to determine by TEM. Middelboe et al. (2003) observed that the viral community in estuarine sediments was dominated by filamentous viruses with a life cycle of chronic infection, which was considered to be an adaptation to the benthic environment. Lammers (1988) observed that over 80% of the prokaryotes isolated from river sediments were lysogens, though Knowles et al. (2017) observed no significant difference in the proportion of lysogens between benthic and pelagic habitats. These factors may lead to the differences in VPR values between benthic and pelagic habitats, resulting in varied ecological effects. Further research is needed to determine the most important factor.

Most of the current understanding of the ecological role of viruses depends on viral lytic infections that occur in pelagic habitats, especially the importance of viruses in organic flows and biogeochemical cycles. The universally low benthic VPR suggests that the virus–host relationship and the ecological function of viruses in benthic habitats may be very different from those of pelagic habitats, and the knowledge obtained from pelagic viruses cannot be directly applied. Viruses may not be as active in benthic habitats as previously thought, especially in nutrient-deficient habitats such as the deep-sea plain (Danovaro et al., 2008a). Thus, greater attention should be paid to the characteristics of benthic viruses and microhabitats to gain a deeper understanding of the ecological functions of benthic viruses.

## Conclusion

The VPR values in sediments has been overestimated due to the use of the sample centrifugation method, which should be avoided in future studies. In surface sediments, VPR values are low and relatively less varied across both temporal and spatial scales. The mean VPR was value approximately 2 in both marine and freshwater habitats, fluctuating mostly within 10. From the intertidal zone to the deep-sea plain, the VPR showed a downward trend, but its relationship with the water depth was not significant. The VPR showed an insignificant seasonal variation trend of lower values at high temperature in the intertidal zone. The sediment Pha content was the most relevant factor regulating the spatial pattern of the VPR, which decreased along with the trophic gradients from the shallow to deep sea areas. The mean VPR value in the pelagic habitats is one order of magnitude higher than that of sediments and fluctuated to a much greater extent, indicating that the virus–host relationship and the ecological function of viruses in the two ecosystems may be very different.

## Data Availability Statement

All datasets generated for this study are included in the article/Supplementary Material.

## Author Contributions

MW and KX conceived and designed the experiments. MW performed the experiments and data collection. MW and KX analyzed the data and wrote the manuscript.

## Conflict of Interest

The authors declare that the research was conducted in the absence of any commercial or financial relationships that could be construed as a potential conflict of interest.
