# Supplementary material for: New Insights Into the Virus-to-Prokaryote Ratio (VPR) in Marine Sediments
Source: Front Microbiol. 2020 May 29;11:1102. doi: 10.3389/fmicb.2020.01102 (PMC7272709; doi:10.3389/fmicb.2020.01102)
Supplement: Supplementary file 2 [file Image_1.pdf]

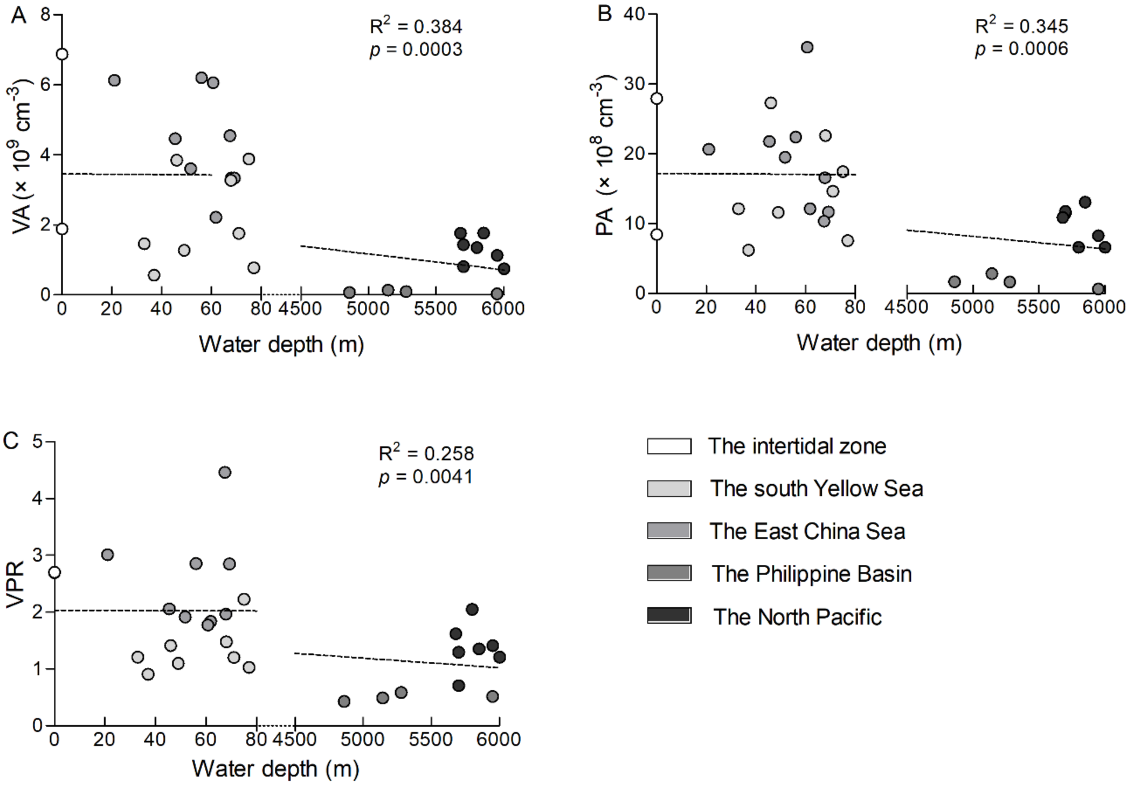

Figure S1. Spatial patterns of sediment **(A)** VA, **(B)** PA and **(C)** VPR along with the water depth gradient in the surveyed areas.
